# Association between red blood cell storage duration and clinical outcome in patients undergoing off-pump coronary artery bypass surgery: a retrospective study

**DOI:** 10.1186/1471-2253-14-95

**Published:** 2014-10-21

**Authors:** Jeong Jin Min, Jun-Yeol Bae, Tae Kyong Kim, Deok Man Hong, Ho Young Hwang, Ki-Bong Kim, Kyou-Sup Han, Yunseok Jeon

**Affiliations:** Department of Anesthesiology and Pain Medicine, Seoul National University Hospital, Seoul, Korea; Department of Anesthesiology and Pain Medicine, Samsung Medical Center, Sungkyunkwan University School of Medicine, Seoul, Korea; Department of Thoracic and Cardiovascular Surgery, Seoul National University Hospital, Seoul, Korea; Department of Laboratory Medicine, Seoul National University Hospital, Seoul, Korea

**Keywords:** RBC storage age, Old stored RBC, Postoperative outcome, Wound complication, Cardiac surgery

## Abstract

**Background:**

Prolonged storage of red blood cells (RBCs) leads to fundamental changes in both the RBCs and the storage media. We retrospectively evaluated the relationship between the RBC age and in-hospital and long-term postoperative outcomes in patients undergoing off-pump coronary artery bypass.

**Methods:**

The electronic medical records of 1,072 OPCAB patients were reviewed and information on the transfused RBCs and clinical data were collected. The effects of RBCs age (mean age, oldest age of transfused RBCs, any RBCs older than 14 days) on various in-hospital postoperative complications and long-term major adverse cardiovascular and cerebral events over a mean follow-up of 31 months were investigated. Correlations between RBCs age and duration of intubation, intensive care unit, or hospital stay, and base excess at the first postoperative morning were also analyzed.

**Results:**

After adjusting for confounders, there was no relationship between the RBCs age and in-hospital and long-term clinical outcomes except for postoperative wound complications. A significant linear trend was observed between the oldest age quartiles of transfused RBCs and the postoperative wound complications (quartile 1 vs. 2, 3 and 4: OR, 8.92, 12.01 and 13.79, respectively; P for trend = 0.009). The oldest transfused RBCs showed significant relationships with a first postoperative day negative base excess (P = 0.021), postoperative wound complications (P = 0.001), and length of hospital stay (P = 0.008).

**Conclusions:**

In patients undergoing off-pump coronary artery bypass, the oldest age of transfused RBCs were associated with a postoperative negative base excess, increased wound complications, and a longer hospital stay, but not with the other in-hospital or long-term outcomes.

**Electronic supplementary material:**

The online version of this article (doi:10.1186/1471-2253-14-95) contains supplementary material, which is available to authorized users.

## Background

Prolonged storage of red blood cells (RBCs) alters them and their storage media, causing changes referred to as ‘storage lesions’. Over time, intracellular adenosine triphosphate within the stored RBCs decreases, rendering the RBC membrane fragile and less deformable [1]. The breakdown of fragile RBCs releases free hemoglobin and microparticles which reduce nitric oxide bioavailability, leading to vasoconstriction, thrombosis, and inflammation [2, 3]. Moreover, depleted 2,3 DPG decreases oxygen delivery to organs [4]. Numerous studies on various patient populations have investigated the clinical impact of RBC storage lesions. However, their impact is still debatable.

Several studies on cardiac surgery patients have investigated the association between clinical outcomes and the storage time of RBCs [5–9]. However, previous studies used heterogeneous populations that included patients who received open heart surgery for valvular heart disease or an on-pump coronary artery bypass. Valvular heart diseases have various cardiac pathophysiologies according to the disease type and severity. Moreover, cardiopulmonary bypass with hypothermia is associated with inflammatory, metabolic, and hematologic responses and various organ injuries, making elucidation of the effects of old stored blood in such patient populations more complex [10]. Although a few studies have investigated the clinical effects of transfusions of old stored blood in patients undergoing coronary artery bypass surgery with regard to vasoconstrictive, thrombotic, and inflammatory effects [3, 5, 7–9, 11], to our knowledge, the effects of stored RBCs have not been investigated exclusively in patients undergoing off-pump coronary artery bypass (OPCAB) surgery.

Although the transfusion rate of RBCs in off-pump CABG surgery was lower than that in on-pump surgery, more than half of the OPCAB patients still needed RBCs transfusion [12]. In patients with coronary arterial disease, RBCs transfusion is essential for adequate oxygen delivery. Meanwhile, old blood transfusion is a concern in such patients because of the possible harmful vascular effects [2, 3].

We hypothesized that prolonged storage of RBCs may be associated with adverse in-hospital and long-term postoperative outcomes in patients undergoing OPCAB. To evaluate this hypothesis, we retrospectively studied the relationship between the RBC storage duration and in-hospital clinical outcomes and long-term postoperative major adverse cardiovascular and cerebral events (MACCEs) in patients undergoing OPCAB.

## Methods

The study protocol was approved by the institutional review board of our hospital (IRB No. 1302-052-465, Seoul National University Hospital). As this was a retrospective study using electronic medical records, individual informed consent was waived. We screened the computerized medical records of 1,113 patients who underwent OPCAB between December 2005 and May 2012 and identified patients who received RBC transfusions during their hospital stay. A total of 41 patients who had not received RBC transfusions were excluded. Therefore, the final study population included 1,072 patients.

### Data collection

The electronic medical records of enrolled patients were reviewed and pre-, intra-, and postoperative data were collected by researchers who were not aware of the RBC transfusion information. The clinical follow-up concluded in September 2012, with a mean follow-up duration of 31 (inter-quartile range [IQR], 11–51) months.

To determine the quantity and age of RBCs, information about all RBC units transfused to enrolled patients during their hospital stay was obtained from the computerized database of our institutional blood bank with the aid of the hospital’s Medical Information Department. The storage time (in days) of RBCs was analyzed in three ways: (1) the mean age of transfused RBCs units, (2) the oldest age of transfused RBCs units, and (3) any transfusion of RBCs units older than 14 days as a categorical variable.

All RBCs units were provided by the Korean Red Cross Blood Services. The RBCs were stored in citrate phosphate dextrose adenine (CPDA)-1 and the storage temperature was 2–6°C. RBCs units in our institution’s blood bank are discarded after 35 days of storage. The perioperative coagulation management strategy was as follows: all patients took aspirin until the day of the surgery and resumed it as soon as possible after the surgery, usually one day postoperatively. During the surgery, the patients were given an initial dose of heparin (1.5 mg/kg) and periodic supplemental doses to maintain an ACT >300 sec. Heparin was neutralized at the end of the surgery to only one-third of the required protamine dose. The perioperative target hemoglobin level was 10 g/dl.

### Study end points and definition

The primary endpoint was the in-hospital and long-term MACCEs, defined as a composite of death from cardiac causes, myocardial infarction (MI), coronary revascularization, and stroke. The long-term follow-up was initiated after hospital discharge and concluded in September 2012. The mean follow-up period was 31 months, with the range 0 to 80 months (median 29, inter-quartile range [IQR], 11–51 months). Other study endpoints were in-hospital postoperative adverse outcomes including all-cause mortality, new renal failure, respiratory complications, postoperative wound complications, a new arrhythmia requiring treatment, bleeding-related reoperations, and the length of ICU and hospital stay. Definitions of each in-hospital postoperative outcome are as follows; Death was considered to be of cardiac origin if attributed to myocardial infarction, cardiac arrhythmia, or heart failure caused primarily by a cardiac problem. MI or stroke diagnosis and coronary revascularization were confirmed by reviewing hospital records. Respiratory complications included prolonged ventilator support (>48 h) or postoperative pneumonia. The diagnosis of pneumonia was based on a combination of physical signs and a chest X-ray and often confirmed by microbiological tests. Postoperative new renal failure was defined as an increase of >50% in serum creatinine from the preoperative value or the requirement for new renal replacement therapy regardless of serum creatinine level. Postoperative wound complication was defined as any sternal wound complication after surgery such as superficial and deep sternal wound including mediastinitis. Arrhythmias other than atrial fibrillation were defined as a postoperative new arrhythmia requiring treatment, including frequent multifocal premature ventricular contractions, ventricular bigemini or quadrigemini, junctional rhythm, paroxysmal supraventricular tachycardia, ventricular tachycardia, ventricular fibrillation and asystole. Bleeding-related reoperation was confirmed by reviewing hospital records.

### Statistical analysis

Continuous variables are presented as the mean (SD) and categorical variables as numbers and percentages. Linearity assumptions in the continuous variables were examined using restricted cubic splines. After checking for violation of the proportional hazard assumption, Cox proportional hazards regression models were used to identify the univariate and multivariable covariates associated with long-term MACCEs. Hazard ratios (HRs) and 95% confidence intervals (CIs) were calculated for each factor using Cox proportional hazards analysis. To assess the independent impact of each risk factor on various postoperative outcomes, univariate and multivariable logistic regression models were constructed.

Variables that included as risk factors for adverse postoperative outcomes were as follows: total number of transfused RBCs, patient’s age, sex, body mass index, presence of diabetes mellitus, hypertension, dyslipidemia, previous history of myocardial infarction, previous history of stroke, presence of renal failure, left ventricular dysfuction (LV ejection fraction less than 35%), chronic obstructive pulmonary disease, cardiac reoperation, perioperative IABP insertion, emergency operation and the duration of surgery. In the Cox regression model for MACCEs, perioperative use of statin, anesthetic agent, lowest values of intraoperative hemodynamic variables and lowest hematocrit were also included as covariates.

All adjusted models were constructed using the forward variable selection method and a forward selection criterion for model fit of *P* =0.1 was used. To determine the effect of RBC age on in-hospital and long-term clinical outcomes, we constructed each adjusted model including each RBC age as a covariate after forward variable selection although they showed insignificant results in the univariate analysis.

Pearson’s correlation or Spearman’s rank correlation coefficients were used as appropriate to analyze the relationships between RBC transfusion amount and some continuous variables such as postoperative base excess, total bilirubin, length of ICU and hospital stay. Partial correlation analyses were used to remove the effects of the number of transfused RBCs in the relationships between the three ages of RBCs and those continuous variables. A *P* value of <0.05 was considered to indicate statistical significance. Analyses were performed using SPSS 19.0 (SPSS, Chicago, IL).

## Results

**Figure 1:**
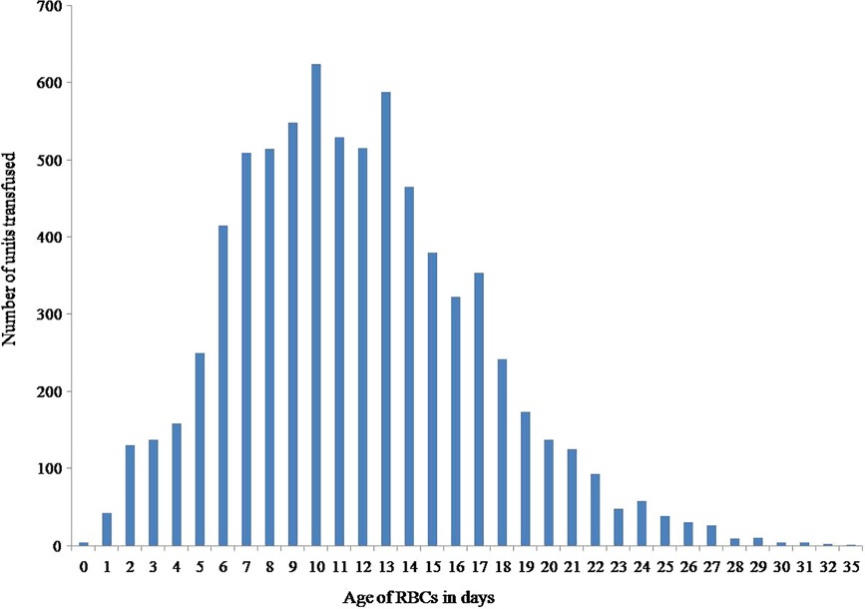
**Storage age of all transfused RBCs.** The figure shows the distribution of RBCs age and the number of transfusions for each RBCs age. RBC, red blood cell.

**Figure 2:**
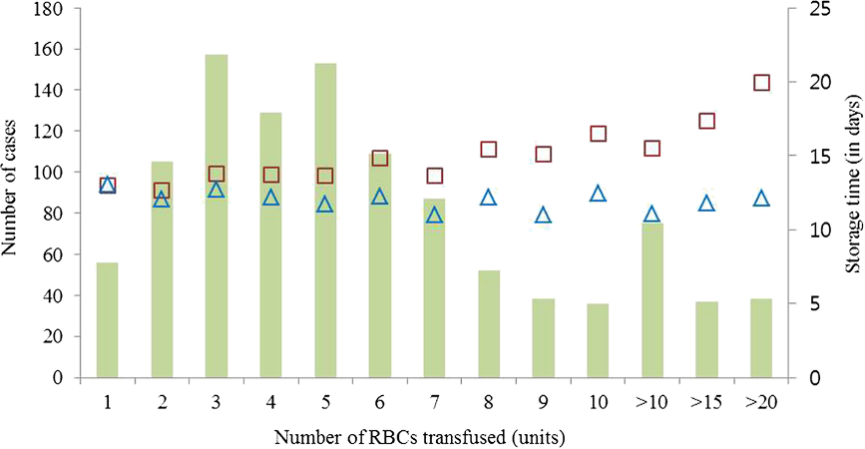
**Transfusion amounts per patient and its relationship with the RBCs age.** Bars represent the number of cases for each transfusion amounts. In each number of transfusions, squares (□) represent the average value of ‘oldest age of transfused RBCs’ and triangles (△) represent the average value of ‘mean age of transfused RBCs’. RBC, red blood cell.

Baseline and operative characteristics of the 1,072 patients studied are shown in Table 1. Although there was a male dominance (71.6%), there was no sex-related difference in the transfused RBCs amounts. A total of 7,480 allogenic, non-leukoreduced RBC units were transfused in the 1,072 patients. The mean storage time of the transfused RBCs was 11.7 (5.2) days (range, 0–35 days); the distribution of the storage time of all transfused RBCs units is shown in Figure 1. The average number of transfused RBCs per patient was 7 (8) units and the distribution of the number of transfused RBCs and the average values of RBCs age according to the number of transfused RBCs units are shown in Figure 2. In all, 473 patients (44%) received at least one RBC unit that was older than 14 days.

**Table 1:** **Baseline and operative characteristics**

Patient variables	
Number of patients	1072
Age, yr	65 (9)
Male	766 (71.5%)
Body mass index (kg m^-2^)	25 (3)
Diabetes mellitus	528 (49.3%)
Hypertension	771 (71.9%)
Dyslipidemia	246 (22.9%)
Previous myocardial infarction	86 (8.0%)
Previous stroke	155 (14.5%)
Acute renal failure	17 (1.6%)
Chronic renal failure	156 (14.6%)
LV dysfunction (EF < 35%)	151 (14.1%)
COPD	12 (1.1%)
Previous cardiac surgery	35 (3.3%)
Preoperative IABP insertion	218 (20.3%)
Operative variables	
Emergent operation	181 (16.9%)
Number of coronary grafts	3 (1)
Duration of surgery, min	370 (77)

Data are presented as mean (SD) or number (%).

LV, left ventricle; EF, ejection fraction; COPD, chronic obstructive pulmonary disease; IABP, intra-aortic balloon pump.

### Long-term MACCEs

MACCEs occurred in 16.9% (181/1072) of patients during the follow-up period, including 15 deaths from cardiac causes, 48 myocardial infarctions, 88 coronary revascularizations, and 44 strokes. Some patients had more than one complication. The detailed description of the components of MACCEs was presented in the Additional file 1. In univariate analysis, MACCEs were significantly associated with the amount of RBCs transfused but not with the storage time of RBCs (Table 2). After adjusting for the variables with *P* <0.1 in the univariate analyses, total number of transfused RBCs (HR, 1.03; 95% CI, 1.02–1.04; P <0.001), and the use of postoperative statin (HR, 0.56; 95% CI, 0.37–0.86; P =0.007) were the factors significantly associated with MACCEs. We constructed separate adjusted models including those two significant factors and the age of RBCs as covariates, which revealed no significant association between RBCs age and long-term MACCEs (Table 2).

**Table 2:** **Predictors for MACCEs**

	Univariable analysis			Adjusted model 1 ^*^			Adjusted model 2 ^†^			Adjusted model 3 ^‡^		
	HR	95% CI	P value	HR	95% CI	P value	HR	95% CI	P value	HR	95% CI	P value
Total number of RBCs transfusion	1.04	1.03-1.05	<0.001	1.03	1.02-1.04	<0.001	1.03	1.02-1.04	<0.001	1.03	1.02-1.04	<0.001
Mean RBCs age	0.97	0.94-1.00	0.053	0.98	0.95-1.01	0.248						
Maximum RBCs age	1.01	0.99-1.04	0.368				1.001	0.97-1.03	0.948			
Any RBCs unit >14 days	1.01	0.75-1.35	0.957							0.89	0.65-1.24	0.504
Age	1.03	1.01-1.04	0.005									
Male	0.94	0.68-1.28	0.679									
Body mass index (kg m^-2^)	0.99	0.95-1.04	0.652									
Smoking	0.92	0.69-1.23	0.563									
Diabetes mellitus	0.9	0.67-1.21	0.493									
Hypertension	1.4	0.99-1.98	0.059									
Dyslipidemia	1.13	0.81-1.58	0.47									
Previous myocardial infarction	1.05	0.62-1.78	0.852									
Previous stroke	1.27	0.87-1.88	0.221									
Acute renal failure	1.97	0.73-5.33	0.179									
Chronic renal failure	1.6	1.1-2.34	0.015									
LV dysfunction (EF < 35%)	1.46	0.99-2.14	0.053									
COPD	2.83	1.05-7.62	0.04									
Previous cardiac surgery	1.24	0.58-2.65	0.572									
Perioperative IABP insertion	1.77	1.30-2.40	<0.001									
Emergent operation	1.59	1.13-2.24	0.008									
Duration of surgery (min)	1	1.00-1.00	0.093									
preoperative statin use	0.88	0.65-1.17	0.35									
postoperative statin use	0.49	0.32-0.75	0.001	0.56	0.37-0.85	0.007	0.56	0.37-0.86	0.007	0.56	0.37-0.85	0.006
anesthetics (sevoflurane vs. propofol)	0.82	0.59-1.15	0.26									
lowest intraoperative heart rate	0.99	0.98-1.01	0.828									
lowest intraoperative mean arterial pressure	1	0.99-1.01	0.87									
lowest intraoperative mixed venous saturation	1.02	0.99-1.04	0.24									
lowest intraoperative cardiac index	0.8	0.48-1.35	0.4									
lowest perioperative hematocrit	0.97	0.92-1.01	0.129									

^*^Adjusted model 1 was constructed including the mean RBCs age as a covariate.

^†^Adjusted model 2 was constructed including the oldest RBCs age as a covariate.

^‡^Adjusted model 3 was constructed including the ‘any RBCs unit >14 days’ as a covariate.

MACCE, major adverse cardiovascular and cerebral event; RBC, red blood cell; LV, left ventricle; EF, ejection fraction; COPD, chronic obstructive pulmonary disease; IABP, intra-aortic balloon pump.

### In-hospital complications

The incidence of each postoperative clinical outcome is presented in Table 3. The oldest transfused RBCs were strongly associated with all of the in-hospital postoperative adverse outcomes except for MACCEs in the univariate analyses but the predictive effects disappeared in multivariable analyses adjusting for confounders, except for postoperative wound complications (Table 3, see also Additional file 2). For postoperative wound complications, oldest age of transfused RBCs (OR, 1.09; 95% CI, 1.04–1.14; P =0.001), total number of transfused RBCs (OR, 1.03; 95% CI, 1.01–1.06; P =0.001), presence of diabetes mellitus (OR, 1.96; 95% CI, 1.05–3.67; P =0.035), and body mass index (OR, 1.14; 95% CI, 1.03–1.25; P =0.008) were significantly associated on multivariable analysis (Additional file 2). The number of transfused RBCs was significantly correlated with all of the analyzed clinical outcomes even after adjusting for confounding parameters (Table 3, Additional file 2). Mean age of transfused RBCs was not significantly related with any of the postoperative clinical outcomes. A significant association between ‘any RBCs unit >14 days’ and some postoperative outcomes (postoperative wound complications and bleeding-related reoperation) disappeared after adjusting for confounders (Table 3).

**Table 3:** **The age or the number of transfused RBCs and the postoperative clinical outcomes**

		Univariate analysis			Multivariable analysis		
	N (%)	OR	95% CI	P value	OR	95% CI	P value
**Number of transfused RBCs**							
In-hospital all-cause mortality	17 (1.6)	1.1	1.07-1.14	<0.001	1.1	1.06-1.14	<0.001
In-hospital MACCEs	74 (6.9)	1.06	1.04-1.09	<0.001	1.05	1.03-1.08	<0.001
New renal failure	54 (5.0)	1.05	1.03-1.07	<0.001	1.03	1.00-1.05	0.04
Respiratory complication	33 (3.1)	1.05	1.03-1.07	<0.001	1.04	1.01-1.06	0.005
Postoperative wound complication	49 (4.6)	1.04	1.02-1.06	<0.001	1.03	1.01-1.06	0.001
Atrial fibrillation	290 (29.1)	1.04	1.02-1.06	<0.001	1.02	1.00-1.04	0.038
Arrhythmia other than atrial fibrillation	112 (10.4)	1.05	1.03-1.07	<0.001	1.03	1.02-1.05	<0.001
Bleeding-related reoperation	29 (2.7)	1.09	1.06-1.12	<0.001	1.08	1.05-1.12	<0.001
**Oldest age of transfused RBCs**							
In-hospital all-cause mortality		1.1	1.02-1.19	0.015	1.01	0.91-1.12	0.896
In-hospital MACCEs		1.01	0.97-1.05	0.63	0.97	0.93-1.02	0.198
New renal failure		1.08	1.03-1.13	0.001	1.03	0.98-1.09	0.251
Respiratory complication		1.07	1.01-1.13	0.02	1.01	0.95-1.08	0.726
Postoperative wound complication		1.1	1.05-1.15	<0.001	1.09	1.04-1.14	0.001
Atrial fibrillation		1.03	1.00-1.05	0.022	1.01	0.99-1.04	0.339
Arrhythmia other than atrial fibrillation		1.04	1.00-1.07	0.037	1.02	0.98-1.05	0.348
Bleeding-related reoperation		1.08	1.02-1.15	0.007	1.02	0.95-1.10	0.531
**Mean age of transfused RBCs**							
In-hospital all-cause mortality		0.93	0.84-1.04	0.23	0.95	0.82-1.09	0.446
In-hospital MACCEs		0.95	0.90-1.00	0.053	0.95	0.90-1.01	0.1
New renal failure		0.99	0.93-1.05	0.684	1.01	0.94-1.08	0.857
Respiratory complication		0.99	0.92-1.06	0.731	0.98	0.90-1.06	0.629
Postoperative wound complication		1.04	0.98-1.10	0.201	1.05	0.99-1.12	0.106
Atrial fibrillation		1	0.97-1.03	0.969	1	0.97-1.04	0.783
Arrhythmia other than atrial fibrillation		0.99	0.95-1.03	0.614	1	0.96-1.05	0.869
Bleeding-related reoperation		1.04	0.96-1.12	0.332	1.08	0.99-1.18	0.094
**Any RBCs unit >14 days**							
In-hospital all-cause mortality		1.83	0.64-4.84	0.225	0.7	0.20-2.45	0.278
In-hospital MACCEs		1.02	0.64-1.64	0.933	0.74	0.44-1.24	0.256
New renal failure		1.5	0.87-2.60	0.148	1.05	0.57-1.97	0.869
Respiratory complication		1.54	0.77-3.09	0.224	1.06	0.50-2.27	0.878
Postoperative wound complication		1.96	1.10-3.50	0.023	1.71	0.93-3.12	0.083
Atrial fibrillation		1.2	0.92-1.58	0.18	1.06	0.80-1.41	0.675
Arrhythmia other than atrial fibrillation		1.41	0.95-2.09	0.085	1.19	0.79-1.80	0.407
Bleeding-related reoperation		2.24	1.05-4.75	0.036	1.21	0.50-2.93	0.669

RBC, red blood cell; MACCE, major adverse cardiovascular and cerebral event.

### ICU and hospital length of stay

Total number of transfused RBCs was significantly related with the duration of ICU stay (*r* =0.47, P <0.001), and hospital stay (*r* =0.58, P <0.001). After controlling for the effects of the number of transfused RBCs, the oldest transfused RBCs were significantly correlated with the length of hospital stay (*r* =0.08, P =0.008) but not with the length of ICU stay (Table 4). There was no significant relationship between the other ages of RBCs and the ICU or hospital length of stay (Table 4).

**Table 4:** **Correlation between the transfused RBCs and intubated time, ICU stay and Hospital stay**

	Transfusion amout		Oldest RBCs age		Mean RBCs age		Any RBCs unit >14 days	
	***r***	P value	***r***	P value	***r***	P value	r	P value
ICU stay	0.47	<0.001	-0.001	0.971	-0.005	0.862	0.001	0.976
Hospital stay	0.58	<0001	0.081	0.008	0.007	0.811	0.059	0.056

RBC, red blood cell; ICU, intensive care unit.

### Postoperative base excess

We evaluated the relationship between RBCs age and base excess on first postoperative morning. After adjusting for the transfusion amount, the oldest transfused RBCs showed a significant negative correlation with the postoperative base excess (r = -0.08, *P* =0.02). There were no significant relationships between the other ages of RBCs and the base excess (with mean age of transfused RBCs: r = -0.03, *P* =0.32; with any RBCs unit >14 days: r = -0.05, *P* =0.16).

### Postoperative total bilirubin

We evaluated the relationship between RBCs age and the postoperative highest total bilirubin as an indicator of hemolysis. Postoperative highest total bilirubin was significantly correlated with the total number of transfused RBCs (r =0.19, P <0.001) and the oldest age of transfused RBCs (r =0.12, P <0.001). However, after adjusting for the transfusion amount, the oldest age of transfused RBCs was not significantly related with the postoperative highest total bilirubin (r =0.06, P =0.056).

### Oldest RBCs age and wound complications

As there was a significant association between the oldest transfused RBCs and postoperative wound complications, we constructed an additional adjusted model for postoperative wound complications including the oldest age of transfused RBCs as quartiles rather than a continuous variable. In the adjusted model, a significant linear trend was observed between the oldest age quartiles of transfused RBCs and postoperative wound complications (quartile 1 vs. 2: OR, 8.92; 95% CI, 1.15–69.05; quartile 1 vs. 3: OR, 12.01; 95% CI, 1.56–92.63; quartile 1 vs. 4: OR, 13.79; 95% CI, 1.82–104.76; P for trend =0.009) (Table 5).

**Table 5:** **Predictors for postoperative wound complication**

	Adjusted model			
	OR	95% CI	P value	P for trend ^*^
DM	1.94	1.04-3.63	0.038	
BMI	1.13	1.03-1.25	0.011	
Number of transfused RBCs	1.04	1.02-1.06	< 0.001	
Oldest age quartile 1 of transfused RBCs^*^ (reference)			0.067	0.009
quartile 2	8.92	1.15-69.05	0.036	
quartile 3	12.01	1.56-92.63	0.017	
quartile 4	13.79	1.82-104.76	0.011	

^*^P for trend refers to linear trend across lowest to highest quartile.

^*^Oldest age quartile 1: oldest age of transfused RBCs unit <10 days (n =218), quartile 2: oldest age of transfused RBCs unit <14 days (n =304), quartile 3: oldest age of transfused RBCs unit <18 days (n =254), quartile 4: oldest age of transfused RBCs unit ≥18 days (n =296).

RBC, red blood cell; DM, diabetes mellitus; BMI, body mass index.

There were two patients with other postoperative infections other than wound and the details of those patients were presented in the Additional file 3. We also calculated the postoperative Sepsis related Organ Failure (SOFA) scores [13] and analyzed the relationship with RBCs ages. The total number of transfused RBCs and the oldest age of the transfused RBCs showed significant correlations with the postoperative highest SOFA scores (see Additional file 4).

## Discussion

In this retrospective study, postoperative in-hospital outcome and long-term MACCEs were associated with the amount of transfused RBC units in patients undergoing OPCAB. After adjusting for confounding factors, the age of transfused RBC units was not associated with long-term MACCEs, but was associated with postoperative wound complications, postoperative day 1 negative base excess, and the length of hospital stay. A linear trend was observed between the oldest age quartiles of transfused RBCs and postoperative wound complications.

Several studies have investigated the clinical effects of RBC storage time in patients undergoing cardiac surgery, including coronary artery bypass grafting (CABG). In a previous retrospective analysis of 3,597 CABG patients, the storage time of transfused RBCs was not a significant predictor of early or late mortality [7] and in other retrospective analyses in CABG patients [5, 8, 14, 15], early postoperative morbidities or major adverse cardiovascular events were not associated with RBCs age using various methods of analysis. The results of the present study are consistent with those earlier results in that the significant adverse effects of older RBCs observed in an unadjusted model disappeared after adjusting for confounding factors. However, our study evaluated the adverse effects of older blood on postoperative wound complications while the previous studies did not.

In contrast to our results, a retrospective study that included 6,002 cardiac surgery patients found that transfusion of RBCs older than 14 days increased in-hospital mortality and mechanical ventilation time, caused various complications, and decreased long-term survival after cardiac surgery [9]. However, several baseline characteristics that were significantly different between the newer and older blood groups (e.g., more left ventricular dysfunction, NYHA class IV, mitral regurgitation, and peripheral vascular disease in the older blood group) may have affected the results. There was no difference in the incidence of sternal wound complications between the two groups in that study; however, approximately half of the patients received transfusions of leukoreduced RBCs and their study population included an unknown ratio of on- and off-pump surgery patients. These differences complicated comparing their results with our results. Another study reported a significant association between RBC storage age and the morbidity and mortality after cardiac surgery [16], but it was voluntarily retracted.

Based on the results of many previous studies of the effects of RBCs storage, a storage time of more than 14 days is considered hazardous; thus, 14 days is often used as the cutoff point for patient grouping [7, 9, 15, 17]. The levels of intracellular 2,3-DPG decline to undetectable levels by storage day 14 [3, 18] but begin to normalize within a few hours of transfusion and are completely restored within 72 h after transfusion [19, 20].

The adverse effects of RBC transfusion on postoperative morbidity and mortality have been demonstrated in numerous studies [6, 21–23]. In this study, the number of perioperative RBCs transfusions was associated with in-hospital and long-term clinical outcomes, consistent with previous studies. On the other hand, the clinical adverse effects of storage time of RBCs disappeared after adjusting for confounding factors including transfusion amount, possibly due to RBCs breakdown that occurs during RBCs processing regardless of RBCs age [24].

A dose-dependent adverse effect of RBCs transfusions on postoperative wound complications has been reported in numerous studies and increased body mass index and diabetes mellitus have been identified as additional risk factors [25–28]; our results are consistent with these findings. Moreover, we found that the oldest age of transfused RBCs was also associated with increased postoperative wound complications. Although it is difficult to determine the causal relationship, significant relationships were observed between the oldest transfused RBCs and the postoperative negative base excess (r = -0.079, P =0.02), between negative base excess and postoperative wound complications (r = -0.086, P =0.01), and between postoperative wound complications and prolonged hospital stay (r =0.28, P <0.001). Monitoring of the intra- and postoperative acid–base status has been used as a surrogate marker of tissue oxygen delivery and cellular perfusion [29]. The negative base excess was significantly correlated with the oldest age of transfused RBCs units, which may be due to insufficient oxygen supply to the tissues. This may suggest that older blood transfusions induce tissue hypoperfusion, which increases postoperative wound complications, consequently leading to a prolonged hospital stay.

Moreover, reduced bioavailability of nitric oxide in older RBCs units might have delayed the wound healing [3]. A number of studies investigated the role of nitric oxide in wound healing [30–33]. Synthesis of nitric oxide occurs during wound healing, especially in the early stage of healing [30], and the released nitric oxide improved wound repair by angiogenesis, collagen formation, cell proliferation, and fibroblast migration in damaged tissue [31–33]. Possible other explanation for increased wound complications was transfusion-related immunomodulation in the blood recipients, especially those receiving non-leukoreduced RBCs [34]. A previous prospective cohort study with trauma patients reported that prestorage leukoreduction abrogated the detrimental effect of old stored blood [35]. Although the different characteristics of the study population make it difficult to compare the findings of that study with our results, leukoreduction might have reduced the wound infection in our study.

This study had several limitations. Our study population contained only coronary disease patients who underwent OPCAB by a single surgeon in a single tertiary center. Therefore, it was relatively more homogeneous than populations in previous studies of cardiac surgery patients. However, the retrospective design means that uncontrolled biases could have affected our analyses. As it is difficult to randomize patients to receive older blood transfusions due to ethical concerns and technical problems, the results of some randomized controlled trials of cardiac surgery patients (ClinicalTrials.gov No.: NCT00458783 and NCT00991341) [36] awaiting publication will be important in drawing conclusions about the effects of old stored RBCs on various clinical outcomes.

For another limitation, all transfused RBCs in this study were from nonleukocyte-depleted blood and the clinical effects of stored leukocyte-depleted or irradiated RBCs remain unclear. Filtered RBCs unit showed different level of RBCs hemolysis and leukocyte breakdown from unfiltered RBCs unit over time [24], and gamma irradiation increases hemolysis of RBCs causing potassium leakage and liberates reactive oxygen species. Thus, further studies are needed to identify the clinical effects of those RBCs unit.

## Conclusions

Postoperative in-hospital outcome and long-term MACCEs were associated with the amount of transfused RBC units. The oldest transfused RBC units were associated with the greatest incidence of postoperative wound complications, negative base excess, and longest duration of hospital stay, but not with the other in-hospital outcomes or long-term MACCEs. This suggests that hypoperfused tissue caused by the transfusion of older stored blood increases the incidence of postoperative wound complications, consequently increasing the hospital stay.

## Electronic supplementary material

Additional file 1: **Detailed description of long-term MACCEs.** (PDF 86 KB)

Additional file 2: **Predictors for postoperative clinical outcome.** (PDF 261 KB)

Additional file 3: **Postoperative infections other than wound.** (PDF 34 KB)

Additional file 4: **Postoperative SOFA score and RBCs age.** (PDF 92 KB)

## Abbreviations

RBC: Red blood cell
OPCAB: Off-pump coronary artery bypass
MACCE: Major adverse cardiovascular and cerebral event
ICU: Intensive care unit
CABG: Coronary artery bypass grafting.

## References

[1] Kim-Shapiro DB, Lee J, Gladwin MT (2011). Storage lesion: role of red blood cell breakdown. Transfusion.

[2] Doctor A, Spinella P (2012). Effect of processing and storage on red blood cell function in vivo. Semin Perinatol.

[3] Roback JD (2011). Vascular effects of the red blood cell storage lesion. Hematology Am Soc Hematol Educ Program.

[4] Van de Watering L (2011). Red cell storage and prognosis. Vox Sang.

[5] Van de Watering L, Lorinser J, Versteegh M, Westendord R, Brand A (2006). Effects of storage time of red blood cell transfusions on the prognosis of coronary artery bypass graft patients. Transfusion.

[6] McKenny M, Ryan T, Tate H, Graham B, Young VK, Dowd N (2011). Age of transfused blood is not associated with increased postoperative adverse outcome after cardiac surgery. Br J Anaesth.

[7] Van Straten AH, Soliman Hamad MA, Van Zundert AA, Martens EJ, Ter Woorst JF, de Wolf AM, Scharnhorst V (2011). Effect of duration of red blood cell storage on early and late mortality after coronary artery bypass grafting. J Thorac Cardiovasc Surg.

[8] Yap CH, Lau L, Krishnaswamy M, Gaskell M, Yii M (2008). Age of transfused red cells and early outcomes after cardiac surgery. Ann Thorac Surg.

[9] Koch CG, Li L, Sessler DI, Figueroa P, Hoeltge GA, Mihaljevic T, Blackstone EH (2008). Duration of red-cell storage and complications after cardiac surgery. N Engl J Med.

[10] Esper SA, Subramaniam K, Tanaka KA (2014). Pathophysiology of cardiopulmonary bypass: current strategies for the prevention and treatment of anemia, coagulopathy, and organ dysfunction. Semin Cardiothorac Vasc Anesth.

[11] Sweeney J, Kouttab N, Kurtis J (2009). Stored red blood cell supernatant facilitates thrombin generation. Transfusion.

[12] Lamy A, Devereaux PJ, Prabhakaran D, Taggart DP, Hu S, Paolasso E, Straka Z, Piegas LS, Akar AR, Jain AR, Noiseux N, Padmanabhan C, Bahamondes JC, Novick RJ, Vaijyanath P, Reddy S, Tao L, Olavegogeascoechea PA, Airan B, Sulling TA, Whitlock RP, Ou Y, Ng J, Chrolavicius S, Yusuf S (2012). Off-pump or on-pump coronary-artery bypass grafting at 30 days. N Engl J Med.

[13] Vincent JL, Moreno R, Takala J, Willatts S, De Mendonca A, Bruining H, Reinhart CK, Suter PM, Thijs LG (1996). The SOFA (Sepsis-related Organ Failure Assessment) score to describe organ dysfunction/failure: on behalf of the working group on sepsis-related problems of the European Society of Intensive Care Medicine. Intensive Care Med.

[14] Vamvakas EC, Carven JH (2000). Length of storage of transfused red cells and postoperative morbidity in patients undergoing coronary artery bypass graft surgery. Transfusion.

[15] Voorhuis FT, Dieleman JM, de Vooght KM, van Dijk D, van Herwerden LA, Peelen LM, van Klei WA (2013). Storage time of red blood cell concentrates and adverse outcomes after cardiac surgery: a cohort study. Ann Hematol.

[16] Basran S, Frumento RJ, Cohen A, Lee S, Du Y, Nishanian E, Kaplan HS, Stafford-Smith M, Bennett-Guerrero E (2006). The association between duration of storage of transfused red blood cells and morbidity and mortality after reoperative cardiac surgery. Anesth Analg.

[17] Weinberg JA, McGwin G, Griffin RL, Huynh VQ, Cherry SA, Marques MB, Reiff DA, Kerby JD, Rue LW (2008). Age of transfused blood: an independent predictor of mortality despite universal leukoreduction. J Trauma.

[18] Almac E, Ince C (2007). The impact of storage on red cell function in blood transfusion. Best Pract Res Clin Anaesthesiol.

[19] Steiner ME, Stowell C (2009). Does red blood cell storage affect clinical outcome? When in doubt, do the experiment. Transfusion.

[20] Kor DJ, Van Buskirk CM, Gajic O (2009). Red blood cell storage lesion. Bosn J Basic Med Sci.

[21] Koch CG, Li L, Duncan AI, Mihaljevic T, Loop FD, Starr NJ, Blackstone EH (2006). Transfusion in coronary artery bypass grafting is associated with reduced long-term survival. Ann Thorac Surg.

[22] Koch CG, Li L, Van Wagoner DR, Duncan AI, Gillinov AM, Blackstone EH (2006). Red cell transfusion is associated with an increased risk for postoperative atrial fibrillation. Ann Thorac Surg.

[23] Koch CG, Li L, Duncan AI, Mihaljevic T, Cosgrove DM, Loop FD, Starr NJ, Blackstone EH (2006). Morbidity and mortality risk associated with red blood cell and blood-component transfusion in isolated coronary artery bypass grafting. Crit Care Med.

[24] Sowemimo-Coker SO (2002). Red blood cell hemolysis during processing. Transfus Med Rev.

[25] Vymazal T, Horacek M, Durpekt R, Hladikova M, Cvachovec K (2009). Is allogeneic blood transfusion a risk factor for sternal dehiscence following cardiac surgery? A prospective observational study. Int Heart J.

[26] Banbury MK, Brizzio ME, Rajeswaran J, Lytle BW, Blackstone EH (2006). Transfusion increases the risk of postoperative infection after cardiovascular surgery. J Am Coll Surg.

[27] Friedman ND, Bull AL, Russo PL, Leder K, Reid C, Billah B, Marasco S, McBryde E, Richards MJ (2007). An alternative scoring system to predict risk for surgical site infection complicating coronary artery bypass graft surgery. Infect Control Hosp Epidemiol.

[28] Andreasen JJ, Dethlefsen C, Modrau IS, Baech J, Schonheyder HC, Moeller JK, Johnsen SP (2011). Storage time of allogeneic red blood cells is associated with risk of severe postoperative infection after coronary artery bypass grafting. Eur J Cardiothorac Surg.

[29] Allen M (2011). Lactate and acid base as a hemodynamic monitor and markers of cellular perfusion. Pediatr Crit Care Med.

[30] Witte MB, Barbul A (2002). Role of nitric oxide in wound repair. Am J Surg.

[31] Schwentker A, Vodovotz Y, Weller R, Billiar TR (2002). Nitric oxide and wound repair: role of cytokines?. Nitric Oxide.

[32] Luo JD, Chen AF (2005). Nitric oxide: a newly discovered function on wound healing. Acta Pharmacol Sin.

[33] Han G, Nguyen LN, Macherla C, Chi Y, Friedman JM, Nosanchuk JD, Martinez LR (2012). Nitric oxide-releasing nanoparticles accelerate wound healing by promoting fibroblast migration and collagen deposition. Am J Pathol.

[34] Vamvakas EC, Blajchman MA (2007). Transfusion-related immunomodulation (TRIM): an update. Blood Rev.

[35] Phelan HA, Eastman AL, Aldy K, Carroll EA, Nakonezny PA, Jan T, Howard JL, Chen Y, Friese RS, Minei JP (2012). Prestorage leukoreduction abrogates the detrimental effect of aging on packed red cells transfused after trauma: a prospective cohort study. Am J Surg.

[36] Steiner ME, Assmann SF, Levy JH, Marshall J, Pulkrabek S, Sloan SR, Triulzi D, Stowell CP (2010). Addressing the question of the effect of RBC storage on clinical outcomes: the Red Cell Storage Duration Study (RECESS) (Section 7). Transfus Apher Sci.

[CR37] The pre-publication history for this paper can be accessed here:http://www.biomedcentral.com/1471-2253/14/95/prepub

