# Supplementary material for: Association between red blood cell storage duration and clinical outcome in patients undergoing off-pump coronary artery bypass surgery: a retrospective study
Source: BMC Anesthesiol. 2014 Oct 21;14:95. doi: 10.1186/1471-2253-14-95 (PMC4210472; doi:10.1186/1471-2253-14-95)
Supplement: Supplementary file 1 — Additional file 1: Detailed description of long-term MACCEs. (PDF 86 KB) [file 12871_2014_306_MOESM1_ESM.pdf]

Additional file 1. Detailed description of long-term MACCEs

MACCEs occurred in 16.9% (181/1072) of patients during the follow-up period, including 15 deaths from cardiac causes, 48 myocardial infarctions (MI), 88 coronary revascularizations, and 44 strokes. Some patients had more than one complication. Among 15 deaths, 2 patients had combined MI. One patient who had revascularization due to MI died. One patient who had MI also had stroke combined. Ten patients had coronary revascularization from MIs.

1) Cardiac deaths

Among the 15 cardiac deaths, 12 of them were expired due to cardiac arrest with fatal ventricular arrhythmia, 1 had fatal STEMI and 2 died from heart failure. Two patients with fatal arrhythmia had combined MIs.

2) Myocardial infarctions

The diagnosis of MI was based on the universal definition of myocardial infarction.[1] Among 48 MI patients, 27 were diagnosed with STEMI. Twenty-two patients had a regional wall motion abnormality on echocardiography, two patients had perfusion defects on cardiac SPECT, 35 patients had an abnormal electrocardiogram (e.g., new Q wave, new LBBB, abnormal ST, etc.), and all MI patients showed a rise and fall in cardiac enzymes (CK-MB, troponin). For the management and treatment strategy, 11 had coronary revascularization (one died), two had an intra-aortic balloon pump inserted, and the others had an intravenous infusion of heparin and nitroglycerine. Two patients who had combined pulmonary edema required intubation and mechanical ventilation support. There were four deaths with three of them were cardiac deaths due to MI.

3) Strokes

There were 44 postoperative strokes. Among 44 strokes, 2 were hemorrhagic strokes and 19 were embolic strokes. Among 19 embolic strokes, 15 occurred during the early postoperative period (within months) and four in the late postoperative period (1 or 2 yrs after the surgery). Ten patients had postoperative onset atrial fibrillation. Twenty-three strokes of undetermined type were classified as either ischemic or embolic infarction,

without intracranial hemorrhage (from a review of their brain imaging).

We analyzed the association between the stroke type and the RBCs age (oldest or mean age of transfused RBCs and any RBCs unit older than 14 days) by Cox proportional hazards analysis, adjusting for the same confounders used in Table 2. The results revealed no significant relationship between each stroke type and the RBC age. However, we could not include the 23 strokes of undetermined type in the analysis, and there was only limited information on stroke outcomes in the medical records.

	Embolic stroke			Hemorrhagic stroke		
	HR	95% CI	P value	HR	95% CI	P value
Mean RBCs age	1.02	0.92-1.12	0.75	1.11	0.80-1.54	0.54
Maximum RBCs age	1.01	0.93-1.10	0.8	1.14	0.82-1.60	0.42
Any RBCs >14 days	1.37	0.52-3.50	0.52	3.27	0.1-105.01	0.5

Reference

1. Thygesen K, Alpert JS, White HD: **Universal definition of myocardial infarction**. *European heart journal* 2007, **28**(20):2525-2538.
