# Supplementary material for: Association between red blood cell storage duration and clinical outcome in patients undergoing off-pump coronary artery bypass surgery: a retrospective study
Source: BMC Anesthesiol. 2014 Oct 21;14:95. doi: 10.1186/1471-2253-14-95 (PMC4210472; doi:10.1186/1471-2253-14-95)
Supplement: Supplementary file 2 — Additional file 2: Predictors for postoperative clinical outcome. (PDF 261 KB) [file 12871_2014_306_MOESM2_ESM.pdf]

Additional file 2. Predictors for postoperative clinical outcome

RBC, red blood cell; LV, left ventricle; EF, ejection fraction; COPD, chronic obstructive pulmonary disease; IABP, intra-aortic balloon pump; MACCE, major adverse cardiovascular and cerebral event;

In-hospital all-cause mortality

	Unadjusted model			Adjusted model 1			Adjusted model 2			Adjusted model 3		
	OR	95% CI	P value	OR	95% CI	P value	OR	95% CI	P value	OR	95% CI	P value
Total number of transfused RBCs	1.1	1.07-1.14	< 0.001	1.09	1.06-1.13	< 0.001	1.09	1.06-1.13	< 0.001	1.1	1.06-1.14	< 0.001
Oldest age of transfused RBCs	1.1	1.02-1.19	0.015	1.01	0.91-1.12	0.896						
Mean age of transfused RBCs	0.93	0.84-1.04	0.23				0.95	0.82-1.09	0.446			
Any RBCs unit > 14 days	1.83	0.69-4.84	0.225							0.7	0.20-2.45	0.278
Age	1.08	1.01-1.15	0.019									
Male	1.3	0.42-4.03	0.645									
Body mass index (kg m^{-2})	0.87	0.74-1.04	0.124									
Diabetes mellitus	1.16	0.45-3.03	0.759									
Hypertension	1.27	0.41-3.94	0.675									
Dyslipidemia	1.41	0.49-4.03	0.525									
Previous myocardial infarction	1.54	0.35-6.85	0.57									
Previous stroke	0.79	0.18-3.47	0.751									
Chronic renal failure	3.29	1.20-9.03	0.021									
LV dysfunction (EF<35%)	3.42	1.25-9.40	0.017									

COPD	5.93	0.72-48.72	0.098									
Previous cardiac surgery	1.88	0.24-14.56	0.547									
Perioperative IABP insertion	8.15	2.84-23.36	< 0.001	3.7	1.11-12.26	0.033	3.72	1.12-12.38	0.032	3.71	1.12-12.32	0.032
Emergent operation	4.53	1.72-11.91	0.002									
Duration of surgery (min)	1.01	1.00-1.01	0.051									

In-hospital MACCE

	Unadjusted model			Adjusted model 1			Adjusted model 2			Adjusted model 3		
	OR	95% CI	P value	OR	95% CI	P value	OR	95% CI	P value	OR	95% CI	P value
Total number of transfused RBCs	1.06	1.04-1.09	<0.001	1.06	1.04-1.09	< 0.001	1.05	1.03-1.08	< 0.001	1.06	1.03-1.08	< 0.001
Oldest age of transfused RBCs	1.01	0.97-1.05	0.63	0.97	0.93-1.02	0.198						
Mean age of transfused RBCs	0.95	0.90-1.00	0.053				0.95	0.90-1.01	0.1			
Any RBCs unit > 14 days	1.02	0.64-1.64	0.933							0.74	0.44-1.24	0.256
Age	1.03	1.01-1.06	0.018									
Male	0.88	0.53-1.46	0.617									
Body mass index (kg m^{-2})	0.98	0.92-1.05	0.631									
Diabetes mellitus	0.64	0.40-1.05	0.075									
Hypertension	1.14	0.67-1.96	0.634									
Dyslipidemia	1	0.57-1.76	0.996									
Previous myocardial infarction	1.43	0.66-3.08	0.362									
Previous stroke	0.92	0.46-1.83	0.811									
Acute renal failure	0.84	0.11-6.43	0.867									
Chronic renal failure	1.15	0.60-2.18	0.674									
LV dysfunction (EF<35%)	1.76	0.99-3.16	0.056									
COPD	2.74	0.59-12.76	0.198									

Previous cardiac surgery	1.28	0.38-4.27	0.693									
Perioperative IABP insertion	3.43	2.12-5.54	<0.001	2.56	1.54-4.26	< 0.001	2.53	1.52-4.22	< 0.001	2.55	1.53-4.25	< 0.001
Emergent operation	1.78	1.03-3.08	0.039									
Duration of surgery (min)	1	1.00-1.006	0.069									

Postoperative new renal failure

	Unadjusted model			Adjusted model 1			Adjusted model 2			Adjusted model 3		
	OR	95% CI	P value	OR	95% CI	P value	OR	95% CI	P value	OR	95% CI	P value
Total number of transfused RBCs	1.05	1.03-1.07	<0.001	1.03	1.00-1.05	0.022	1.03	1.01-1.06	0.005	1.03	1.01-1.06	0.007
Oldest age of transfused RBCs	1.08	1.03-1.13	0.001	1.03	0.98-1.09	0.251						
Mean age of transfused RBCs	0.99	0.93-1.05	0.684				1.01	0.94-1.08	0.857			
Any RBCs unit > 14 days	1.5	0.87-2.60	0.148							1.05	0.57-1.97	0.869
Age	1.05	1.02-1.09	0.003									
Male	2.38	1.11-5.10	0.026									
Body mass index (kg m^{-2})	0.97	0.88-1.06	0.475									
Diabetes mellitus	2.56	1.41-4.65	0.002									
Hypertension	2.73	1.22-6.10	0.015									
Dyslipidemia	0.85	0.43-1.68	0.644									
Previous myocardial infarction	1.18	0.46-3.05	0.732									
Previous stroke	1.96	1.02-3.74	0.043									
Chronic renal failure	18.11	9.80-33.50	<0.001	14.82	7.78-28.20	< 0.001	15.23	8.02-28.92	< 0.001	15.17	7.98-28.81	< 0.001
LV dysfunction (EF<35%)	3.33	1.84-6.03	<0.001	2.23	1.13-4.43	0.022	2.24	1.13-4.44	0.021	2.24	1.13-4.43	0.021
COPD	6.6	1.73-25.1	0.006	6.85	1.27-36.86	0.025	6.73	1.27-35.53	0.025	6.67	1.26-35.23	0.026

Previous cardiac surgery	1.15	0.27-4.92	0.852
Perioperative IABP insertion	1.54	0.84-2.86	0.166
Emergent operation	2.62	1.45-4.73	0.001
Duration of surgery (min)	1	1.00-1.01	0.03

Postoperative respiratory complication

	Unadjusted model			Adjusted model 1			Adjusted model 2			Adjusted model 3		
	OR	95% CI	P value	OR	95% CI	P value	OR	95% CI	P value	OR	95% CI	P value
Total number of transfused RBCs	1.05	1.03-1.07	<0.001	1.05	1.02-1.07	< 0.001	1.05	1.02-1.07	< 0.001	1.05	1.02-1.07	< 0.001
Oldest age of transfused RBCs	1.07	1.01-1.13	0.02	1.01	0.95-1.08	0.726						
Mean age of transfused RBCs	0.99	0.92-1.06	0.731				0.98	0.90-1.06	0.629			
Any RBCs unit > 14 days	1.54	0.77-3.09	0.224							1.06	0.50-2.27	0.878
Age	1.11	1.06-1.16	<0.001	1.08	1.03-1.14	0.002	1.08	1.03-1.14	0.002	1.08	1.03-1.14	0.002
Male	1.5	0.64-3.50	0.347									
Body mass index (kg m^{-2})	0.87	0.77-0.99	0.03									
Diabetes mellitus	1.41	0.70-2.85	0.334									
Hypertension	1.23	0.55-2.75	0.619									
Dyslipidemia	0.33	0.1-1.08	0.067	0.23	0.06-0.91	0.035	0.23	0.06-0.89	0.034	0.23	0.06-0.90	0.034
Previous myocardial infarction	2.66	1.07-6.64	0.036									
Previous stroke	1.06	0.40-2.78	0.909									
Acute renal failure	10.89	3.35-35.42	<0.001									
Chronic renal failure	4.07	1.98-8.37	<0.001	2.39	1.10-5.21	0.028	2.41	1.11-5.23	0.027	2.4	1.10-5.24	0.027
LV dysfunction (EF<35%)	4.85	2.38-9.90	<0.001	3.16	1.47-6.79	0.003	3.16	1.47-6.79	0.003	3.17	1.47-6.82	0.003

COPD	6.64	1.40-31.58	0.017
Previous cardiac surgery	2.66	1.07-6.64	0.036
Perioperative IABP insertion	1.49	0.68-3.25	0.318
Emergent operation	2.56	1.22-5.37	0.013
Duration of surgery (min)	1	0.99-1.01	0.787

Postoperative atrial fibrillation

	Unadjusted model			Adjusted model 1			Adjusted model 2			Adjusted model 3		
	OR	95% CI	P value	OR	95% CI	P value	OR	95% CI	P value	OR	95% CI	P value
Total number of transfused RBCs	1.04	1.02-1.06	<0.001	1.02	1.00-1.04	0.016	1.02	1.01-1.04	0.008	1.02	1.01-1.04	0.01
Oldest age of transfused RBCs	1.03	1.00-1.05	0.022	1.01	0.99-1.04	0.339						
Mean age of transfused RBCs	1	0.97-1.03	0.969				1	0.97-1.04	0.783			
Any RBCs unit > 14 days	1.2	0.92-1.58	0.18							1.06	0.80-1.41	0.675
Age	1.05	1.04-1.07	<0.001	1.04	1.03-1.06	< 0.001	1.04	1.03-1.06	< 0.001	1.04	1.03-1.06	< 0.001
Male	1.35	0.99-1.84	0.056	1.61	1.16-2.23	0.004	1.62	1.17-2.25	0.004	1.62	1.17-2.24	0.004
Body mass index (kg m^{-2})	0.98	0.93-1.02	0.347									
Diabetes mellitus	1.01	0.77-1.33	0.925									
Hypertension	1.69	1.23-2.33	0.001	1.72	1.22-2.42	0.002	1.71	1.21-2.41	0.002	1.71	1.12-2.40	0.002
Dyslipidemia	0.92	0.66-1.27	0.598									
Previous myocardial infarction	1.84	1.16-2.89	0.009	1.77	1.09-2.87	0.02	1.76	1.09-2.86	0.022	1.76	1.09-2.86	0.022
Previous stroke	1.62	1.13-2.33	0.008									
Acute renal failure	2.4	0.92-6.29	0.074									
Chronic renal failure	2.09	1.47-2.97	<0.001									
LV dysfunction (EF<35%)	1.59	1.10-2.29	0.013									

COPD	1.92	0.60-6.08	0.271									
Previous cardiac surgery	1.07	0.51-2.25	0.867									
Perioperative IABP insertion	1.77	1.31-2.40	<0.001	1.56	1.13-2.16	0.007	1.57	1.13-2.16	0.007	1.56	1.13-2.16	0.007
Emergent operation	1.68	1.20-2.35	0.003									
Duration of surgery (min)	1	1.00-1.00	0.025									

Postoperative arrhythmia other than atrial fibrillation

	Unadjusted model			Adjusted model 1			Adjusted model 2			Adjusted model 3		
	OR	95% CI	P value	OR	95% CI	P value	OR	95% CI	P value	OR	95% CI	P value
Total number of transfused RBCs	1.05	1.03-1.07	<0.001	1.03	1.01-1.05	0.001	1.03	1.01-1.05	< 0.001	1.03	1.01-1.05	0.001
Oldest age of transfused RBCs	1.04	1.00-1.07	0.037	1.02	0.98-1.05	0.348						
Mean age of transfused RBCs	0.99	0.95-1.03	0.614				1	0.96-1.05	0.869			
Any RBCs unit > 14 days	1.41	0.95-2.09	0.085							1.19	0.79-1.80	0.407
Age	1.04	1.02-1.07	0.001	1.03	1.01-1.06	0.008	1.03	1.01-1.06	0.007	1.03	1.01-1.06	0.007
Male	1.16	0.74-1.81	0.512									
Body mass index (kg m^{-2})	0.98	0.91-1.05	0.496									
Diabetes mellitus	0.88	0.60-1.31	0.528									
Hypertension	1.08	0.69-1.67	0.748									
Dyslipidemia	1.33	0.85-2.06	0.21									
Previous myocardial infarction	1.44	0.76-2.74	0.27									
Previous stroke	1.15	0.67-1.97	0.608									
Acute renal failure	1.15	0.26-5.08	0.858									
Chronic renal failure	1.32	0.79-2.21	0.296									
LV dysfunction (EF<35%)	2.02	1.25-3.26	0.004									

COPD	1.73	0.37-7.99	0.484									
Previous cardiac surgery	0.51	0.12-2.16	0.361									
Perioperative IABP insertion	3.29	2.20-4.93	< 0.001	2.68	1.76-4.09	< 0.001	2.69	1.77-4.10	< 0.001	2.69	1.76-4.09	< 0.001
Emergent operation	2.53	1.64-3.92	<0.001									
Duration of surgery (min)	1	1.00-1.01	0.061									

Postoperative wound complication

	Unadjusted model			Adjusted model 1			Adjusted model 2			Adjusted model 3		
	OR	95% CI	P value	OR	95% CI	P value	OR	95% CI	P value	OR	95% CI	P value
Total number of transfused RBCs	1.04	1.02-1.06	<0.001	1.03	1.01-1.06	0.001	1.04	1.02-1.07	< 0.001	1.04	1.02-1.06	<0.001
Oldest age of transfused RBCs	1.1	1.05-1.15	<0.001	1.09	1.04-1.14	0.001						
Mean age of transfused RBCs	1.04	0.98-1.10	0.201				1.05	0.99-1.12	0.106			
Any RBCs unit > 14 days	1.96	1.10-3.50	0.023							1.71	0.93-3.12	0.083
Age	1.01	0.98-1.05	0.47									
Male	0.56	0.31-1.01	0.054									
Body mass index (kg m^{-2})	1.11	1.01-1.22	0.026	1.14	1.03-1.25	0.008	1.12	1.02-1.24	0.015	1.13	1.03-1.24	0.014
Diabetes mellitus	2.07	1.14-3.76	0.017	1.96	1.05-3.67	0.035	2.01	1.08-3.75	0.029	2	1.07-3.74	0.029
Hypertension	0.88	0.47-1.64	0.686									
Dyslipidemia	0.86	0.42-1.74	0.665									
Previous myocardial infarction	0.74	0.23-2.43	0.618									
Previous stroke	1.77	0.88-3.54	0.108									
Acute renal failure	1.31	0.17-10.09	0.795									
Chronic renal failure	0.95	0.42-2.16	0.91									
LV dysfunction (EF<35%)	1.4	0.66-2.94	0.38									

COPD	1.92	0.24-15.15	0.537
Previous cardiac surgery	1.28	0.30-5.48	0.743
Preoperative IABP insertion	1.29	0.66-2.51	0.461
Emergent operation	1.28	0.63-2.61	0.501
Duration of surgery (min)	1	0.99-1.01	0.502

Postoperative bleeding-related reoperation

	Unadjusted model			Adjusted model 1			Adjusted model 2			Adjusted model 3		
	OR	95% CI	*P* value	OR	95% CI	*P* value	OR	95% CI	*P* value	OR	95% CI	*P* value
Total number of transfused RBCs	1.09	1.06-1.12	<0.001	1.11	1.07-1.15	< 0.001	1.12	1.08-1.15	< 0.001	1.11	1.07-1.15	< 0.001
Oldest age of transfused RBCs	1.08	1.02-1.15	0.007	1.02	0.95-1.10	0.531						
Mean age of transfused RBCs	1.04	0.96-1.12	0.332				1.08	0.99-1.18	0.094			
Any RBCs unit > 14 days	2.24	1.05-4.75	0.036							1.21	0.50-2.93	0.669
Age	1.02	0.98-1.06	0.411									
Male	1.95	0.74-5.15	0.179									
Body mass index (kg m^{-2})	0.92	0.80-1.04	0.186									
Diabetes mellitus	0.62	0.29-1.33	0.22									
Hypertension	1.9	0.72-5.03	0.195									
Dyslipidemia	1.29	0.56-2.95	0.548									
Previous myocardial infarction	0.85	0.20-3.62	0.821									
Previous stroke	1.24	0.47-3.30	0.666									
Acute renal failure	2.29	0.29-17.89	0.429									
Chronic renal failure	1.91	0.80-4.55	0.144									
LV dysfunction (EF<35%)	1.28	0.48-3.41	0.621									
COPD	7.65	1.60-36.62	0.011									

Previous cardiac surgery	2.27	0.52-9.94	0.278									
Perioperative IABP insertion	2.96	1.42-6.14	0.004									
Emergent operation	1.92	0.84-4.40	0.125									
Duration of surgery (min)	1.01	1.00-1.01	0.001	1.01	1.00-1.01	0.037	1.01	1.00-1.01	0.031	1.01	1.00-1.01	0.038
