# Supplementary material for: Association between red blood cell storage duration and clinical outcome in patients undergoing off-pump coronary artery bypass surgery: a retrospective study
Source: BMC Anesthesiol. 2014 Oct 21;14:95. doi: 10.1186/1471-2253-14-95 (PMC4210472; doi:10.1186/1471-2253-14-95)
Supplement: Supplementary file 3 — Additional file 3: Postoperative infections other than wound. (PDF 34 KB) [file 12871_2014_306_MOESM3_ESM.pdf]

Additional file 3. Postoperative infections other than wound

Two patients were diagnosed with catheter-related infections, confirmed by blood culture. Both had diabetic end-stage renal disease (ESRD) and were undergoing periodic hemodialysis. One patient was discharged after antibiotics therapy for enterococcal bacteremia (*E. faecalis* was isolated from four blood samples: two peripheral and two central veins, Case 1). Another patient who had gram-positive bacteremia, postoperative pneumonia, and a diabetic foot infection died from septic shock, despite receiving antibiotics therapy (Case 2). Information on the RBCs transfused into the two patients are presented in the table below.

	Case 1	Case 2
Transfused RBCs amount (unit)	18	19
Oldest age of transfused RBCs (d)	13	20
Mean age of transfused RBCs (d)	6.4	14.7
Any RBCs unit older than 14 days	0	1
