# Supplementary material for: Association between red blood cell storage duration and clinical outcome in patients undergoing off-pump coronary artery bypass surgery: a retrospective study
Source: BMC Anesthesiol. 2014 Oct 21;14:95. doi: 10.1186/1471-2253-14-95 (PMC4210472; doi:10.1186/1471-2253-14-95)
Supplement: Supplementary file 4 — Additional file 4: Postoperative SOFA score and RBCs age. (PDF 92 KB) [file 12871_2014_306_MOESM4_ESM.pdf]

Additional file 4. Postoperative SOFA score and RBCs age

We calculated the postoperative Sepsis related Organ Failure (SOFA) scores [1] and analyzed the relationship with RBCs ages. The total number of transfused RBCs and the oldest age of the transfused RBCs showed significant correlations with the postoperative highest SOFA scores.

	r	*P*-value
Amount of transfused RBCs	0.43	<0.001
Oldest age of transfused RBCs	0.18	<0.001
after removing the effect of the transfusion amount	0.1	0.002
Mean age of transfused RBCs	0.003	0.933
after removing the effect of the transfused amount	0.03	0.39

Reference

1. Vincent JL, Moreno R, Takala J, Willatts S, De Mendonca A, Bruining H, Reinhart CK, Suter PM, Thijs LG: **The SOFA (Sepsis-related Organ Failure Assessment) score to describe organ dysfunction/failure. On behalf of the Working Group on Sepsis-Related Problems of the European Society of Intensive Care Medicine**. *Intensive care medicine* 1996, **22**(7):707-710.
